# *Helicobacter pylori* infection and apolipoprotein B/apolipoprotein A1 ratio: a cross-sectional study

**DOI:** 10.3389/fcimb.2025.1582843

**Published:** 2025-05-20

**Authors:** Chao Liu, Xuping Zhu, Jiale Pu, Zhuoqun Zou, Lu Zhou, Xiaowei Zhu

**Affiliations:** ^1^ Department of Endocrinology and Metabolism, The Affiliated Wuxi People’s Hospital of Nanjing Medical University, Wuxi People’s Hospital, Wuxi Medical Center, Nanjing Medical University, Wuxi, Jiangsu, China; ^2^ Department of Geriatrics, Shanghai Health and Medical Center, Wuxi, Jiangsu, China; ^3^ Department of Surgery, Shanghai Health and Medical Center, Wuxi, Jiangsu, China

**Keywords:** *Helicobacter pylori*, lipid metabolism abnormalities, ApoB/ApoA1 ratio, cross-sectional studies, cardiovascular disease

## Abstract

**Background:**

*Helicobacter pylori* (HP) infection is one of the most common chronic infections worldwide, closely related to various gastrointestinal diseases and metabolic disorders. In recent years, the relationship between HP infection and abnormal glucose and lipid metabolism has received significant attention, although its specific mechanisms remain unclear. This study aims to explore the association between HP infection and lipid metabolism abnormalities, particularly the role of the apolipoprotein B/A1 (ApoB/ApoA1) ratio.

**Methods:**

This cross-sectional study retrospectively analyzed data from 9,218 patients who underwent physical examinations at Shanghai Health and Medical Center in 2022. HP infection status was determined using the carbon-13 breath test, and clinical data, biochemical indicators, and lipid metabolism-related data were collected. Multiple regression analysis was employed to investigate the relationship between HP infection and the ApoB/ApoA1 ratio.

**Results:**

Patients in the HP-positive group were older and had a higher proportion of males. Their body mass index (BMI), blood pressure, γ-glutamyl transpeptidase (γ-GT), total cholesterol (TC), fasting blood glucose (FBG), Creatinine and White blood Cell were significantly higher than those in the HP-negative group. The HP-positive group exhibited a higher prevalence of underlying diseases (e.g., hypertension, diabetes, coronary heart disease) and significant abnormalities in glucose and lipid metabolism, uric acid, high-sensitivity C-reactive protein (hs-CRP), and other indicators. The ApoB/ApoA1 ratio was significantly elevated in the HP-positive group and was not influenced by gender. Multiple regression analysis revealed that the ApoB/ApoA1 ratio is an independent risk factor for HP infection.

**Conclusion:**

HP infection is closely associated with abnormal lipid metabolism, and the ApoB/ApoA1 ratio is an independent risk factor for HP infection, demonstrating significant advantages over other lipid indicators. This large-scale study highlights a significant association between HP infection and an elevated ApoB/ApoA1 ratio. The findings suggest that HP may contribute to cardiovascular risk via dyslipidemia, with the ApoB/ApoA1 ratio serving as a potential biomarker. Further research should explore whether HP eradication could mitigate these metabolic disturbances.

## 1 Introduction

*Helicobacter pylori* (HP) is a microaerophilic, spiral-shaped, Gram-negative bacterium that colonizes the stomach. HP infection is one of the most common chronic infections globally, affecting over 50% of the world’s population, with approximately 4.4 billion individuals infected (Li et al., 2017). HP infection is a well-established independent risk factor for gastrointestinal diseases such as peptic ulcers, chronic gastritis, gastric adenocarcinoma, and mucosa-associated lymphoid tissue lymphoma. Recently, attention has shifted to the correlation between HP infection and metabolic disorders. Studies have shown that HP infection is closely associated with impaired glucose and lipid metabolism, including diabetes, obesity, dyslipidemia, atherosclerosis, and nonalcoholic fatty liver disease (Bravo et al., 2018).

Several studies have reported that patients with HP infection exhibit adverse lipid profiles that promote atherosclerosis (Moretti et al., 2014; Zhao et al., 2019; Abdu et al., 2020; Martín-Núñez et al., 2020; Lu et al., 2022). Following treatment, blood lipid levels have been shown to improve after HP eradication with oral antibiotics (Ando et al., 2006). However, the relationship between HP infection and lipid metabolism remains unclear. For instance, Negussie et al. found that total cholesterol (TC) and low-density lipoprotein cholesterol (LDL-C) were elevated in HP-infected patients (Sarbecha et al., 2024), while Sun et al. reported no significant differences in TC and LDL-C levels between HP-infected and non-infected individuals, with HP infection only showing a significant correlation with high-density lipoprotein cholesterol (HDL-C) and triglycerides (TG) (Sun et al., 2016). Xiao et al. observed that TC, TG, and LDL-C levels were higher in the HP-infected group, while HDL-C levels were lower compared to the control group (Yuliandari and Mayura, 2024). Although these findings suggest that HP-infected patients exhibit adverse lipid profiles, the correlation between HP infection and abnormal lipid metabolism varies across studies. Whether other lipid indicators offer potential advantages warrants further investigation.

Apolipoproteins, synthesized in the liver, play a crucial role in lipid transport and redistribution. Apolipoproteins primarily consist of apolipoprotein B (ApoB) and apolipoprotein A1 (ApoA1). The measurement of ApoB and ApoA1 does not require fasting samples and is standardized, making the ApoB/ApoA1 ratio a more balanced, comprehensive, and stable indicator of lipid metabolism and a predictor of cardiovascular disease and diabetes (Jing et al., 2014; Nurtazina et al., 2020). Apolipoproteins also play a significant role in immune regulation and inflammatory responses (Zhong et al., 2022). However, no studies have yet reported a correlation between HP infection and apolipoproteins.

In this study, we conducted a cross-sectional investigation to explore the differences in glucose and lipid metabolism disorders between HP-positive and HP-negative patients, the correlation between HP infection and the ApoB/ApoA1 ratio, and to further elucidate the potential relationship between them. This large-scale study highlights a significant association between HP infection and an elevated ApoB/ApoA1 ratio. The findings suggest that HP may contribute to cardiovascular risk via dyslipidemia, with the ApoB/ApoA1 ratio serving as a potential biomarker. Further research should explore whether HP eradication could mitigate these metabolic disturbances.

## 2 Subjects and methods

### 2.1 Research design

This cross-sectional study was conducted at the Shanghai Health and Medical Center in Wuxi, China. The primary objective was to evaluate the correlation between carbon-13 breath test results and ApoA1, ApoB, and ApoB/ApoA1 ratio.

We collected data from subjects who underwent physical examinations at the Shanghai Health Care Center in 2022. Subjects were screened based on inclusion and exclusion criteria, and general information (e.g., age, gender, height, weight, blood pressure, heart rate), medical history (e.g., diabetes, hypertension, coronary heart disease, vascular plaque), routine biochemical examinations (e.g., white blood cell count, neutrophil count, hemoglobin, high-sensitivity C-reactive protein (hs-CRP), fasting blood glucose (FBG), Hemoglobin A1c, creatinine (HbA1c), alanine aminotransferase (ALT),aspartate aminotransferase (AST),γ-glutamyl transpeptidase (γ-GT), TG, cholesterol, apolipoproteins), and C13 breath test results were collected. Patients were grouped based on C13 breath test results, and intergroup differences were evaluated. Further subgroup analysis by gender was conducted to assess potential influencing factors. Finally, the correlation between C13 breath test results and ApoA1, ApoB, and ApoB/ApoA1 ratio was evaluated.

The confirmation of fatty liver comes from abdominal color Doppler ultrasound. The main diagnostic features include enhanced echo in the front field of the liver (“bright liver”), attenuated echo in the far field, and unclear display of the pipeline structure in the liver to diagnose fatty liver.

The confirmation and classification of carotid atherosclerosis comes from cervical vascular color Doppler ultrasound. Smooth carotid artery: Smooth carotid intima, uniform thickness, IMT less than 1.0mm, and enlargement less than 1.2mm. Coarse carotid artery: The intima of the carotid artery is not smooth, with intermittent echoes or localized thickening of the intima, and an IMT greater than 1.0mm. Thickening of carotid intima: The IMT is between 1.0-1.5mm, and the vascular wall is thickened, but no plaque is formed. Formation of carotid artery plaque: When the IMT is greater than or equal to 1.5mm, there may be plaques protruding into the lumen on the local vascular wall. The plaques can be hypoechoic, mixed echoic, or calcified echoic, and may be accompanied by ulcers or irregular shapes.

### 2.2 Inclusion and exclusion criteria

Inclusion criteria:

1. Age ≥ 18 years.
2. Male or female.

Exclusion criteria:

1. History of gastrointestinal surgery.
2. Antibiotics were used in the last 4 weeks before the examination, regardless of the type and duration of use.
3. Long-term antibiotic use.
4. Alanine aminotransferase or aspartate aminotransferase ≥ 3 times the upper limit of normal.
5. No clear C13 breath test results.
6. Patients with mental, speech, or behavioral disorders.

### 2.3 Study population

This study retrospectively analyzed data from 36,845 patients who underwent physical examinations at Shanghai Health and Medical Center in 2022. Among them, 19,592 patients who did not undergo the C13 breath test were excluded, along with 286 patients with a history of gastrointestinal surgery, malignant tumors, or long-term antibiotic use, 112 patients with elevated liver enzymes, and 7,637 patients with missing data. A total of 9,218 patients were included in the study.

### 2.4 Statistical analysis

Continuous variables with normal distribution are presented as mean ± standard deviation, while non-normally distributed variables are presented as median and interquartile range. Categorical data are presented as frequencies and percentages. One-way analysis of variance (ANOVA) was used to compare clinical features between groups, the Bonferroni-corrected Mann-Whitney U test was used for non-normally distributed variables, and the chi-square test was used for categorical variables. Stepwise multiple regression analysis was conducted to identify independent risk factors, including major clinical risk factors and variables with p-values ≤ 0.1 in univariate analysis. Regression results are expressed as odds ratios (OR) with 95% confidence intervals (CI).

## 3 Results

### 3.1 Baseline characteristics of HP-positive and HP-negative groups

Compared to the HP-negative group, the HP-positive group was older and had a higher proportion of males. Additionally, the HP-positive group had significantly higher BMI, blood pressure, and other indicators. The prevalence of underlying diseases (e.g., hypertension, diabetes, coronary heart disease, vascular plaque) was higher in the HP-positive group, with statistically significant differences. Liver and kidney function, harmful lipid metabolism, glucose metabolism indicators, uric acid, sh-CRP, and white blood cell were also higher in the HP-positive group. Significant differences were observed in ApoB and the ApoB/ApoA1 ratio (p < 0.001 for both). HDL-C and Apo A1 levels were lower in the HP-positive group (p < 0.001 for both), with statistically significant differences (**Table 1**).

**Table 1:** Baseline characteristics of HP-positive and HP-negative groups.

Characteristics	HP-negative	HP-positive	*P*	Characteristics	HP-negative	HP-positive	*P*
N	7308	1910		ALT (U/L)	20 (14, 29)	20 (15, 31)	***0.003***
Age (years)	47 (39, 56)	47 (39, 55)	*0.695*	AST (U/L)	17 (14, 21)	17 (14, 22)	***0.041***
Sex (Male%)	63.10%	69.30%	***0.001***	γ-GT(U/L)	22 (14, 37)	24 (15, 41)	***<0.001***
Height (cm)	167.5 (161.5, 173.4)	168.5 (162.5, 174)	***0.001***	TG (mmol/L)	1 (0.8, 1)	1 (0.8, 1)	***<0.001***
Weight (kg)	68.6 (59.6, 77.1)	71.2 (61.8, 79.5)	***<0.001***	TC (mmol/L)	4.9 (4.4, 5.6)	5 (4.4, 5.6)	***0.011***
BMI (kg/m2)	24.3 (22.2, 26.4)	24.9 (22.9, 27.2)	***<0.001***	LDL-C (mmol/L)	3.1 (2.5, 3.6)	3.1 (2.6, 3.7)	***<0.001***
Systolic pressure (mm/Hg)	122 (111, 132)	124 (113, 135)	***<0.001***	HDL-C (mmol/L)	1.3 (1.1, 1.6)	1.3 (1.1, 1.5)	***<0.001***
Diastolic pressure (mm/Hg)	74 (67, 81)	75 (68, 83)	***<0.001***	ApoA1 (g/L)	1.4 (1.3, 1.6)	1.4 (1.2, 1.5)	***<0.001***
Heart rate (bpm)	76 (68, 84)	76 (69, 84)	***0.003***	ApoB (g/L)	1 (0.8, 1.1)	1 (0.8, 1.2)	***<0.001***
				ApoB/ApoA1 ratio	1.5 (1.2, 1.8)	1.4 (1.1, 1.7)	***<0.001***
Basic medical history				HbA1c (%)	5.7 (5.5, 5.9)	5.7 (5.5, 6)	*0.063*
Hypertension (%)	23.2%	25.6%	***0.031***	FBG (mmol/L)	5.2 (4.9, 5.6)	5.3 (5, 5.7)	***<0.001***
Diabetes (%)	13.3%	15.7%	***0.006***	FINS (pmol/L)	62.3 (43.3, 90.6)	63.9 (44.5, 96)	***0.006***
Fatty liver (%)	44.8%	49.9%	***0.001***	Uric acid (umol/L)	344 (286, 405)	350 (293, 411)	***0.003***
Coronary heart disease (%)	4.5%	5.6%	***0.047***	Creatinine (umol/L)	72.6 (59.9, 83.2)	74 (62.1, 84.6)	***0.001***
Carotid artery ultrasound results (%)				White blood cell (10^9/L)	6 (5.1, 7)	6.3 (5.4, 7.3)	***<0.001***
Smooth carotid artery	62.5%	60.5%	***0.018***	Neutrophils (10^9/L)	3.3 (2.7, 4)	3.5 (2.9, 4.3)	***<0.001***
Coarse carotid artery	13.6%	11.3%		Hemoglobin (g/L)	149 (136, 159)	152 (139, 160)	***<0.001***
Thickening of carotid intima	7.1%	7.7%		Platelet (10^9/L)	237 (205, 274)	239 (204, 275.3)	*0.249*
Formation of carotid artery plaques	16.9%	20.4%		hs-CRP (mg/L)	0.7 (0.3, 1.7)	0.8 (0.3, 1.9)	*0.087*

Data are presented as mean ± standard deviation or median (IQR) or n (%). BMI, Body mass index; ALT, Alanine aminotransferase; AST, Aspartate aminotransferase; γ-GT, γ-glutamyl transpeptidase; TG, Triglyceride; TC, Total cholesterol; LDL-C, Low-density lipoprotein cholesterol; HDL-C, High-density lipoprotein cholesterol; ApoA1, Apolipoprotein A1; ApoB, Apolipoprotein B; HbA1c, Hemoglobin A1c; FBG, Fasting blood glucose; FINS, Fasting insulin; hs-CRP, Hypersensitive C-reactive protein. The bold part in the table represents statistics, P < 0.05, with statistical significance.

### 3.2 Clinical characteristics differences between HP-positive and HP-negative in different gender groups

In the male group, BMI, blood pressure, diabetes history and fatty liver disease history differed significantly between HP-positive and HP-negative patients. In the female group, only BMI showed a significant difference (**Table 2A**). Regardless of gender, LDL-C, HDL-C, ApoA1, ApoB, ApoB/ApoA1 ratio, and white blood cells differed significantly between HP-positive and HP-negative patients. In the male group, γ-GT, TG, FBG, and HbA1c levels also differed significantly (p<0.05), while these differences were not observed in the female group (**Table 2B**).

**Table 2A:** Clinical characteristics differences between HP-positive and HP-negative in different gender groups.

Characteristics	Male			Female		
	HP-negative	HP-positive	*P*	HP-negative	HP-positive	*P*
N	4611	1324		2697	586	
Age (years)	49 (40, 57)	48 (40, 56)	*0.216*	44 (36, 53)	45 (37, 54)	*0.231*
Height (cm)	171.5 (167.5, 175.5)	171.5 (167.5, 175.6)	*0.814*	160 (156.5, 163.6)	159.9 (156, 163.5)	*0.261*
Weight (kg)	74 (67.9, 81.1)	75.6 (69.1, 82.7)	***<0.001***	57.7 (52.9, 63.3)	58.2 (53.7, 64.2)	***0.023***
BMI (kg/m2)	25.2 (23.4, 27.1)	25.6 (23.7, 27.8)	***<0.001***	22.5 (20.7, 24.7)	22.9 (21, 25.2)	***0.002***
Systolic pressure (mm/Hg)	126 (117, 135)	127 (118, 136)	***0.003***	113 (104, 125)	114 (104, 127)	*0.172*
Diastolic pressure (mm/Hg)	76 (69, 83)	77 (70, 84)	***<0.001***	70 (63, 77)	70 (64, 79)	*0.076*
Heart rate (bpm)	75 (68, 83)	75 (69, 84)	***0.015***	76 (70, 84)	78 (70, 85)	***0.026***
Basic medical history						
Hypertension (%)	29.30%	39.40%	*0.432*	12.90%	14.70%	*0.241*
Diabetes (%)	16.90%	19.30%	***0.043***	7.20%	7.70%	*0.658*
Fatty liver (%)	57.50%	60.60%	***0.039***	23.10%	25.60%	*0.203*
Coronary heart disease (%)	5.50%	6.70%	*0.083*	2.90%	3.10%	*0.815*
Carotid artery ultrasound results (%)						
Smooth carotid artery	56.90%	55.00%	*0.097*	75.50%	77.60%	*0.570*
Coarse carotid artery	14.50%	12.30%		11.40%	8.40%	
Thickening of carotid intima	8.10%	8.60%		4.90%	5.20%	
Formation of carotid artery plaques	20.60%	24.00%		8.20%	8.80%	

Data are presented as mean ± standard deviation or median (IQR) or n (%). BMI, Body mass index. The bold part in the table represents statistics, P < 0.05, with statistical significance.

**Table 2B:** Clinical characteristics differences between HP-positive and HP-negative in different gender groups.

Characteristics	Male			Female		
	HP-negative	HP-positive	*P*	HP-negative	HP-positive	*P*
N	4611	1324		2697	586	
ALT (U/L)	23 (17, 33)	23 (17, 34)	*0.368*	15 (11, 20)	15 (11, 21)	*0.761*
AST (U/L)	18 (15, 23)	18 (15, 23)	*0.439*	15 (13, 19)	15 (13, 19)	*0.721*
γ-GT(U/L)	28 (19, 45)	30 (20, 47)	***0.020***	14 (11, 20)	14 (10, 21)	*0.830*
TG (mmol/L)	1 (1, 2)	1 (1, 2)	***0.005***	0.9 (0.6, 1)	0.9 (0.7, 1)	*0.258*
TC (mmol/L)	5 (4.4, 5.6)	5 (4.4, 5.6)	*0.056*	4.9 (4.4, 5.5)	5 (4.4, 5.6)	*0.122*
LDL-C (mmol/L)	3.1 (2.6, 3.7)	3.2 (2.6, 3.7)	***0.019***	3 (2.4, 3.5)	3 (2.5, 3.6)	***0.030***
HDL-C (mmol/L)	1.2 (1.1, 1.4)	1.2 (1, 1.4)	***<0.001***	1.6 (1.3, 1.8)	1.5 (1.3, 1.8)	***0.012***
ApoA1 (g/L)	1.3 (1.2, 1.5)	1.3 (1.2, 1.5)	***0.004***	1.5 (1.4, 1.7)	1.5 (1.4, 1.7)	***0.033***
ApoB (g/L)	1 (0.9, 1.2)	1 (0.9, 1.2)	***<0.001***	0.9 (0.7, 1.1)	0.9 (0.8, 1.1)	***0.016***
ApoB/ApoA1 ratio	1.3 (1.1, 1.7)	1.3 (1.1, 1.6)	***<0.001***	1.7 (1.4, 2.1)	1.7 (1.3, 2)	***0.009***
HbA1c (%)	5.7 (5.5, 6)	5.7 (5.5, 6.1)	*0.101*	5.6 (5.4, 5.8)	5.6 (5.4, 5.8)	*0.392*
FBG (mmol/L)	5.3 (5, 5.8)	5.4 (5.1, 5.8)	***<0.001***	5.1 (4.8, 5.4)	5.1 (4.9, 5.5)	*0.094*
FINS (pmol/L)	67.1 (46.1, 96.6)	68.1 (46.1, 102)	*0.125*	55 (39.1, 79.7)	59 (40.4, 83)	*0.107*
Uric acid (umol/L)	382 (334, 430)	383 (333, 437)	*0.826*	279 (242, 321)	282 (240, 321)	*0.748*
Creatinine (umol/L)	80.4 (73, 88)	80.3 (72.4, 88.2)	*0.401*	57.1 (51.9, 62.5)	57.4 (51.8, 63.4)	*0.749*
White blood cell (10^9/L)	6.2 (5.3, 7.2)	6.5 (5.5, 7.5)	***<0.001***	5.6 (4.8, 6.6)	5.8 (5, 6.9)	***0.001***
Neutrophils (10^9/L)	3.4 (2.8, 4.2)	3.6 (3, 4.4)	***<0.001***	3.1 (2.5, 3.8)	3.2 (2.7, 4)	***0.001***
Hemoglobin (g/L)	156 (150, 162)	157 (150, 163)	***0.040***	133 (127, 139)	133 (125, 139)	*0.148*
Platelet (10^9/L)	230 (200, 264)	233 (200, 267)	*0.152*	251 (215, 288)	256 (217, 296)	*0.073*
hs-CRP (mg/L)	0.8 (0.4, 1.8)	0.9 (0.4, 2.1)	*0.054*	0.5 (0.2, 1.4)	0.5 (0.2, 1.4)	*0.320*

Data are presented as mean ± standard deviation or median (IQR) or n (%). ALT, Alanine aminotransferase; AST, Aspartate aminotransferase; γ-GT, γ-glutamyl transpeptidase; TG, Triglyceride; TC, Total cholesterol; LDL-C, Low-density lipoprotein cholesterol; HDL-C, High-density lipoprotein cholesterol; ApoA1, Apolipoprotein A1; ApoB, Apolipoprotein B; HbA1c, Hemoglobin A1c; FBG, Fasting blood glucose; FINS, Fasting insulin; hs-CRP, Hypersensitive C-reactive protein. The bold part in the table represents statistics, P < 0.05, with statistical significance.

### 3.3 Characteristics of HP-positive and HP-negative in different groups

**Figure 1:**
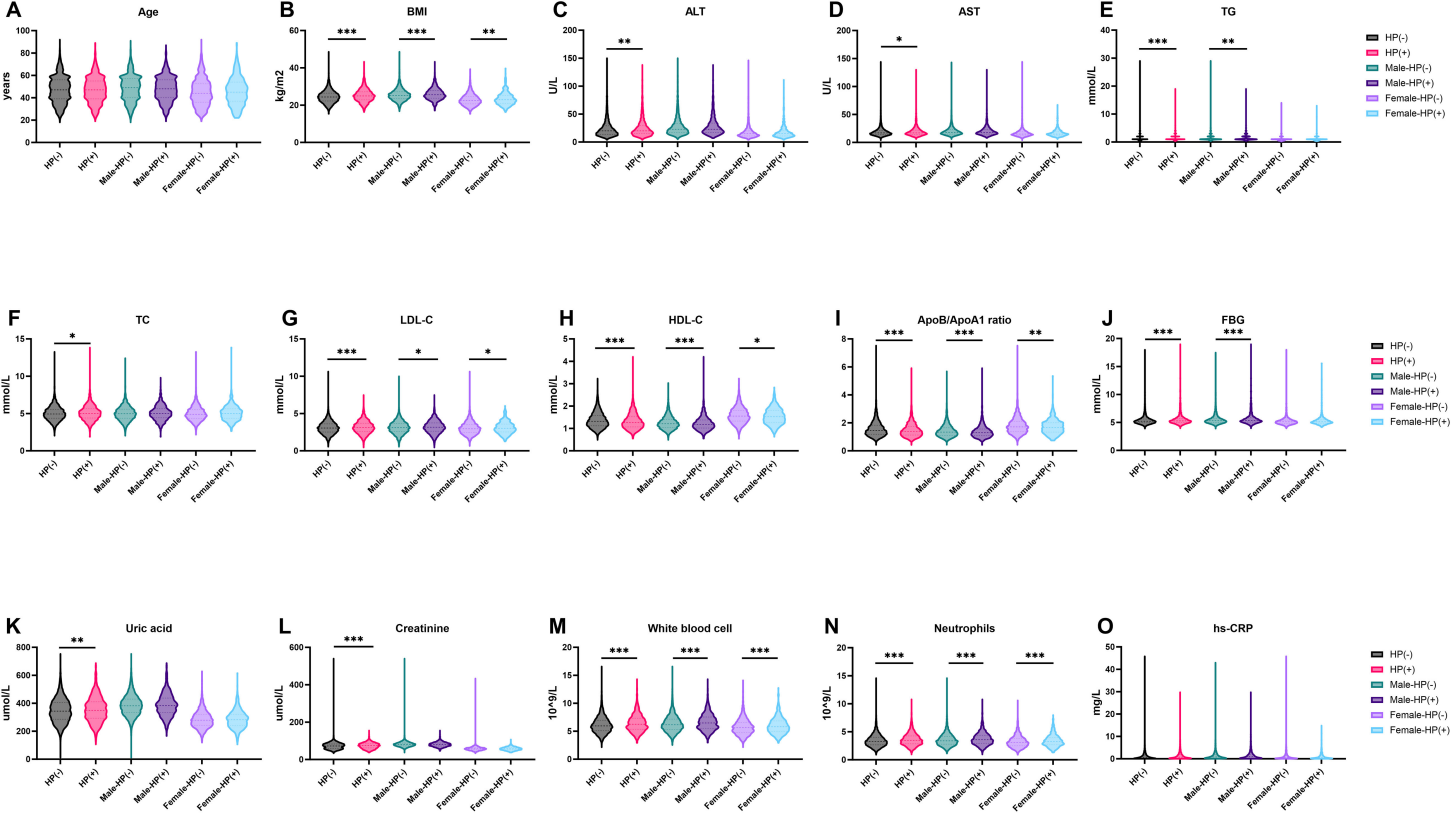
Characteristics of HP-Positive and HP-Negative in different groups. **(A, B) ** show the distribution characteristics of Age and BMI in different groups; **(C–I) ** show the distribution characteristics of ALT, AST, TG, TC, LDL-C, HDL-C and ApoB/ApoA1ratio in different groups; (J-O) show the distribution characteristics of FBG, Uric acid, Creatine, White blood cell, Neutrophils and hs-CRP in different groups. *P < 0.05, **P < 0.01, ***P < 0.001. BMI, Bodymass index; ALT, Alanine aminotransferase; AST, Aspartate aminotransferase; TG, Triglyceride; TC, Total cholesterol; LDL-C, Low-density lipoprotein cholesterol; HDL-C, High-density lipoprotein cholesterol; ApoA1, ApolipoproteinA1; ApoB, ApolipoproteinB; FBG, Fastingbloodglucose; hs-CRP, HypersensitiveC-reactiveprotein.

After grouping by gender, there are still statistical differences in BMI, LDL-C, HDL-C, ApoB/ApoA 1 ratio, white blood cell and neutrophils between HP negative and positive patients (**Figure 1**). The differences in ALT, AST, TC, Uric acid and Creatinine between HP negative and HP positive patients before grouping by gender disappeared after grouping by gender (**Figure 1**).

### 3.4 Single factor logistic regression analysis of HP infection

In the male group, BMI, heart rate, LDL-C, HDL-C, ApoB, ApoB/ApoA1 ratio, FBG, γ-GT, white blood cells, neutrophils, and hs-CRP were significant risk factors for HP positivity (p < 0.05) (**Table 3**). In the female group, BMI, heart rate, HDL-C, ApoB, ApoB/ApoA1 ratio, white blood cells, and neutrophils were significant risk factors for HP positivity (p < 0.05) (**Table 3**).

**Table 3:** Single factor logistic regression analysis of HP infection.

Characteristics	Male					Female				
	*β*	*OR*	*95% CI*		*P*	*β*	*OR*	*95% CI*		*P*
Heart rate(bpm)	0.006	1.006	1.000	1.011	***0.035***	0.009	1.009	1.001	1.017	***0.026***
BMI(kg/m2)	0.044	1.045	1.026	1.065	***<0.001***	0.050	1.052	1.023	1.081	***<0.001***
TG(mmol/L)	0.033	1.033	0.992	1.076	*0.111*	0.037	1.037	0.934	1.152	*0.496*
TC(mmol/L)	0.072	1.075	1.008	1.147	***0.028***	0.064	1.067	0.974	1.168	*0.166*
LDL-C(mmol/L)	0.090	1.094	1.017	1.177	***0.016***	0.095	1.100	0.992	1.220	*0.072*
HDL-C(mmol/L)	-0.350	0.704	0.569	0.872	***0.001***	-0.341	0.711	0.554	0.912	***0.007***
ApoA1(g/L)	-0.352	0.703	0.519	0.952	***0.023***	-0.438	0.645	0.436	0.955	***0.028***
ApoB(g/L)	0.455	1.576	1.231	2.019	***<0.001***	0.393	1.481	1.036	2.117	***0.031***
ApoB/ApoA1 ratio	-0.292	0.746	0.648	0.860	***<0.001***	-0.255	0.775	0.656	0.915	***0.003***
FBG(mmol/L)	0.084	1.087	1.040	1.137	***<0.001***	0.064	1.066	0.968	1.175	*0.196*
γ-GT(U/L)	0.002	1.002	1.001	1.003	***0.007***	-0.002	0.998	0.993	1.003	*0.426*
White blood cell(10^9/L)	0.121	1.129	1.085	1.175	***<0.001***	0.107	1.112	1.048	1.181	***0.001***
Neutrophils(10^9/L)	0.143	1.154	1.095	1.217	***<0.001***	0.128	1.137	1.051	1.230	***0.001***
hs-CRP(mg/L)	0.013	1.013	0.998	1.028	*0.085*	-0.026	0.974	0.932	1.019	*0.256*

Beta is the standardized coefficient, which measures the influence degree of each variable; OR is the odds ratio, which refers to the independent risk; CI is confidence interval. BMI, Body mass index; γ-GT, γ-glutamyl transpeptidase; TG, Triglyceride; TC, Total cholesterol; LDL-C, Low-density lipoprotein cholesterol; HDL-C, High-density lipoprotein cholesterol; ApoA1, Apolipoprotein A1; ApoB, Apolipoprotein B; HbA1c, Hemoglobin A1c; FBG, Fasting blood glucose; hs-CRP, Hypersensitive C-reactive protein. The bold part in the table represents statistics, P < 0.05, with statistical significance.

### 3.5 HP positive ratio in different lipids after quartile division

After dividing TC, LDL-C, HDL-C, ApoA 1, ApoB and ApoB/ApoA 1 ratio into quartiles, the results showed that the positive ratio of *Helicobacter pylori* patients was different, with a linear trend, which was statistically significant (**Table 4**). With the gradual increase of TC, LDL-C and ApoB from Q1 to Q4, the positive rate of patients with *Helicobacter pylori* gradually increased, with statistical significance (p<0.05). With the increase of HDL-C, ApoA 1 and ApoB/ApoA 1 ratio, the positive rate of patients with *Helicobacter pylori* gradually decreased, with statistical significance (p<0.05).

**Table 4:** HP positive ratio in different lipids after quartile division.

Lipid metabolism indicators	Q1	Q2	Q3	Q4	*p*	*p for linear trend*
TC	24.03%	23.30%	25.86%	26.81%	***0.041***	***0.011***
LDL-C	22.83%	24.55%	25.55%	27.07%	***0.018***	***0.002***
HDL-C	30.05%	26.39%	22.15%	21.41%	***<0.001***	***<0.001***
ApoA1	28.90%	25.97%	23.19%	21.94%	***<0.001***	***<0.001***
ApoB	21.88%	24.19%	27.12%	26.81%	***<0.001***	***<0.001***
ApoB/ApoA1 ratio	29.32%	25.86%	23.66%	21.15%	***<0.001***	***<0.001***

Chi-square test was used.TC, Total cholesterol; LDL-C, Low-density lipoprotein cholesterol; HDL-C, High-density lipoprotein cholesterol; ApoA1, Apolipoprotein A1; ApoB, Apolipoprotein B. The bold part in the table represents statistics, P < 0.05, with statistical significance.

### 3.6 Multivariate logistic regression analysis of HP infection

In male patients, FBG, white blood cells, ApoB/ApoA1 ratio, and BMI were independent risk factors for HP positivity (p < 0.05), with OR values of 1.065, 1.106, 0.84, and 1.029, respectively (**Table 5**). In female patients, white blood cells, ApoB/ApoA1 ratio, BMI, and hs-CRP were independent risk factors for HP positivity (p < 0.05), with OR values of 1.113, 0.818, 1.047, and 0.918, respectively (**Table 5**).

**Table 5:** Multivariate logistic regression analysis of HP infection.

Gender	Characteristics	*β*	*OR*	*95% CI*		*P*
**Male**	FBG(mmol/L)	0.063	1.065	1.018	1.114	***0.007***
	White blood cell(10^9/L)	0.1	1.106	1.061	1.152	***<0.001***
	ApoB/ApoA1 ratio	-0.175	0.84	0.727	0.97	***0.018***
	BMI(kg/m2)	0.028	1.029	1.008	1.049	***0.006***
	Constant	-2.733	0.065			***<0.001***
**Female**	White blood cell(10^9/L)	0.107	1.113	1.044	1.186	***0.001***
	ApoB/ApoA1 ratio	-0.2	0.818	0.687	0.975	***0.025***
	BMI(kg/m2)	0.046	1.047	1.016	1.08	***0.003***
	hs-CRP(mg/L)	-0.085	0.918	0.868	0.972	***0.003***
	Constant	-2.769	0.063			***<0.001***

Beta is the standardized coefficient, which measures the influence degree of each variable; OR is the odds ratio, which refers to the independent risk; CI is confidence interval. BMI, Body mass index; ApoA1, Apolipoprotein A1; ApoB, Apolipoprotein B; FBG, Fasting blood glucose; hs-CRP, Hypersensitive C-reactive protein. The bold part in the table represents statistics, P < 0.05, with statistical significance.

## 4 Discussion

This study found that HP infection is closely associated with multiple clinical parameters. The prevalence of underlying diseases, such as hypertension, diabetes, coronary heart disease, and vascular plaque, was higher in the HP-positive group. Significant differences were observed in glucose and lipid metabolism-related indicators, uric acid, hs-CRP, and white blood cells between the HP-positive and HP-negative groups, consistent with previous studies (Bravo et al., 2018; Park et al., 2021; Feng et al., 2024; Yang et al., 2024). These findings suggest that HP-positive individuals may experience systemic glucose and lipid metabolism disorders and chronic inflammation, with HP infection closely linked to cardiovascular system damage.

Multiple pathways connect HP infection and lipid metabolism disorders. The chronic low-grade inflammation induced by HP infection leads to the accumulation of pro-inflammatory cytokines, such as C-reactive protein, interleukin-6, and interleukin-8, exacerbating oxidative stress and lipid metabolism disturbances (Chen et al., 2023). Inflammatory signals activate nuclear factor kappa B (NF-κB), downregulating lipid metabolism-related genes and enhancing pro-inflammatory gene expression (Shi et al., 2022; Wang et al., 2022; Chen et al., 2024). These factors significantly impact host lipid metabolism and atherosclerosis progression, increasing cardiovascular risk (Robinson et al., 2015; Krupa et al., 2021). Additionally, HP infection modulates host energy metabolism by upregulating mTORC1 expression and altering branched-chain amino acid metabolism (Kim et al., 2018; Feng et al., 2020). HP infection also affects metabolic hormone secretion (e.g., ghrelin, leptin, GLP-1) and induces host inflammatory responses by altering gut microbiota composition, impacting host metabolism and energy homeostasis (Cuomo et al., 2020). Conversely, lipid metabolism disorders can affect gastric blood circulation and mucosal barrier function, creating favorable conditions for the colonization and reproduction of Hp. They can also lead to changes in the lipid composition of gastric mucosa, affecting mucosal integrity and permeability, making it easier for Hp to invade the submucosal layer and aggravating the degree of infection (Feng et al., 2020). In addition, lipid metabolism disorders can lead to an imbalance in immune regulation, resulting in a decrease in the body’s immune defense against HP.

Our study also revealed gender differences in clinical characteristics. Male HP-positive patients exhibited significant differences in age, medical history, liver function, lipid metabolism, glucose metabolism, uric acid, and hs-CRP compared to HP-negative males. In contrast, female HP-positive patients showed differences in only a few indicators, which seems to suggest that women themselves have an inhibitory effect on HP induced glucose and lipid metabolism disorders. Limited research exists on gender differences in HP infection.

Previous studies have found a decrease in Hp serum positivity among individuals using contraceptive pills, suggesting a potential protective effect of oral contraceptives against *Helicobacter pylori* infection (Fong and Wang, 2021). Previous epidemiological studies and animal models have shown that female hormones, especially estrogen, have a protective effect on Hp induced gastric cancer, and this study further reveals that *Helicobacter pylori* cells adsorbed with estrogen may be prevented from adhering to gastric epithelial cells, thereby exerting a protective effect (Hosoda et al., 2009). Animal studies have demonstrated that estrogen supplementation alleviates gastric damage in HP-infected male mice by enhancing IL-10 function and reducing IFN-γ and IL-1β responses (Ohtani et al., 2011). Research has found that female mice undergoing ovariectomy develop Hp-induced gastric cancer, while female mice undergoing ovariectomy can inhibit the occurrence of gastric cancer by supplementing with estrogen. Estrogen therapy can alleviate gastric lesions by increasing the expression of Foxp3+ and interleukin-10 (IL-10), reducing the expression of IFN-e and IL-1e (Sheh et al., 2011). At the same time, studies have found that G protein coupled estrogen receptors prevent the activation of NF-κB promoter by Hp cytotoxin related gene A in gastric cancer cells, and inhibit the expression of tumor necrosis factor alpha (TNF-T), IL-6, and IL-1 (Kang et al., 2021; Okamoto et al., 2023),. These studies all suggest that female estrogen has the ability to inhibit inflammation and downstream pathway activation caused by Helicobacter pylori. However, there is no direct research report on whether estrogen is related to glucose and lipid metabolism disorders in Hp, which also deserves further investigation.

Our study further identified through multiple regression analysis that, regardless of gender, BMI, ApoB/ApoA1 ratio, and white blood cells are independent risk factors for HP infection. The ApoB/ApoA1 ratio was significantly elevated in HP-positive individuals and demonstrated superiority over other lipid indicators, a finding not previously reported.

Apolipoproteins play a crucial role in immune regulation and inflammatory responses. During acute infection, ApoA1 inhibits neutrophil phagocytosis and reactive oxygen species production. It also affects the inflammatory response and apoptosis of neutrophils through various pathways, such as activating the AMPK pathway and regulating NF-κF signaling pathways (Meng et al., 2024). While ApoB is a strong inflammatory marker positively correlated with IL-6 and CRP (Zhong et al., 2022). Apolipoproteins, in addition to serving as structural components of lipoproteins, also act as ligands for cell surface receptors and cofactors for enzymes, playing a crucial role in microbial invasion of host cells. The interaction between serum lipids and host cell membrane lipid rafts may alter HP-host cell interactions, leading to chronic inflammation and increased cardiovascular risk (Oakley et al., 2009; Majkova et al., 2010; Kumar et al., 2015; Chyuan et al., 2018). Therefore, the increase of ApoB/ApoA1 is related to chronic inflammation *in vivo*. It is speculated that Hp infection may lead to the increase of ApoB/ApoA1 by causing the occurrence and development of inflammation in the host, and the increase of ApoB/ApoA1 is involved in the interaction between Hp and host cells, and mediates the further progress of inflammation, ultimately leading to atherosclerosis and increased risk of cardiovascular and cerebrovascular diseases. However, the mechanisms underlying the interaction between HP infection and apolipoproteins remain unclear and warrant further investigation.

### 4.1 Limitations

This study has several limitations. As a cross-sectional study, it lacks control variables and long-term follow-up, potentially introducing recall bias, detection bias, and information bias. As well, the ratio of the HP-positive and HP-negative groups is 1: 3 to 1: 4, which may affect the testing efficiency to some extent. Additionally, the study did not collect data on smoking and alcohol consumption, limiting the ability to analyze their potential roles in HP infection or metabolic abnormalities.

## 5 Conclusion

In summary, our study demonstrates that HP infection is closely associated with lipid metabolism abnormalities. The ApoB/ApoA1 ratio is an independent risk factor for HP infection, unaffected by gender, and offers significant advantages over other lipid indicators.

## Data Availability

The raw data supporting the conclusions of this article will be made available by the authors, without undue reservation.
